# Supplementary material for: Adhesion Forces of Oral Bacteria to Titanium and the Correlation with Biophysical Cellular Characteristics
Source: Bioengineering (Basel). 2022 Oct 17;9(10):567. doi: 10.3390/bioengineering9100567 (PMC9598062; doi:10.3390/bioengineering9100567)
Supplement: Supplementary file 1 [file bioengineering-09-00567-s001.zip › bioengineering-1852405-supplementary.pdf]

Supplementary Information

Adhesion Forces of Oral Bacteria to Titanium and the Influence of Cellular Characteristics

Katharina Doll[1,4]*, Andreas Winkel[1,4], Ines Yang[1,4], Anna Josefine Grote[1,4], Nils Meier[2], Mosaieb Habib[3,4,5], Henning Menzel[2], Peter Behrens[3,4,5], Meike Stiesch[1,4]

1 Department of Prosthetic Dentistry and Biomedical Materials Science, Hannover Medical School, Carl-Neuberg-Str. 1, 30625 Hannover, Germany
2 Institute for Technical Chemistry, Technische Universität Braunschweig, Hagenring 30, 38106 Braunschweig, Germany
3 Institute of Inorganic Chemistry, Leibniz University Hannover, Callinstr. 9, 30167 Hannover, Germany
4 Lower Saxony Centre for Biomedical Engineering, Implant Research and Development (NIFE), Stadtfelddamm 34, 30625 Hannover, Germany
5 Cluster of Excellence Hearing4All, Hannover, Germany
* Correspondence: doll.katharina@mh-hannover.de

Table S1. Parameter settings for analysis of force-distance curves from single cell force spectroscopy using the AtomicJ software.[1]

Parameter	Setting
**Processing – Automatic**	
Contact estimator	Classical golden
Estimation method	based on contact model
Model fit	Classical (L2)
Fit to	Withdraw (for maximum adhesion, attachment points, and detachment distance)
	Approach (for Young's modulus)
**Model**	
Model	Sphere (JKR)
Radius (µm)	0.375 (for *S. oralis, P. gingivalis* and *A. ac*)
	0.5 (for *A. naeslundii*)
	0.5 (for *V. dispar*)
**Sample**	
Poisson ratio	0.5
Adhesive energy	Calculate from fit
**Calibration**	
Spring (N/m)	Read-in
Sensitivity (µm/V)	Read-in

[1] Hermanowicz, P.; Sarna, M.; Burda, K.; Gabrys, H. AtomicJ: An Open Source Software for Analysis of Force Curves Review of Scientific Instruments 2014, 85, 063703.

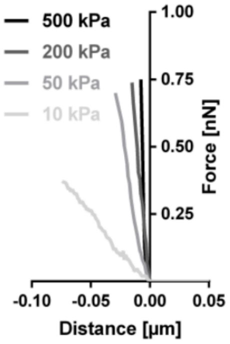

Figure S1. Representative force distance curves for indicated Young's moduli measured as bacterial cell elasticity.

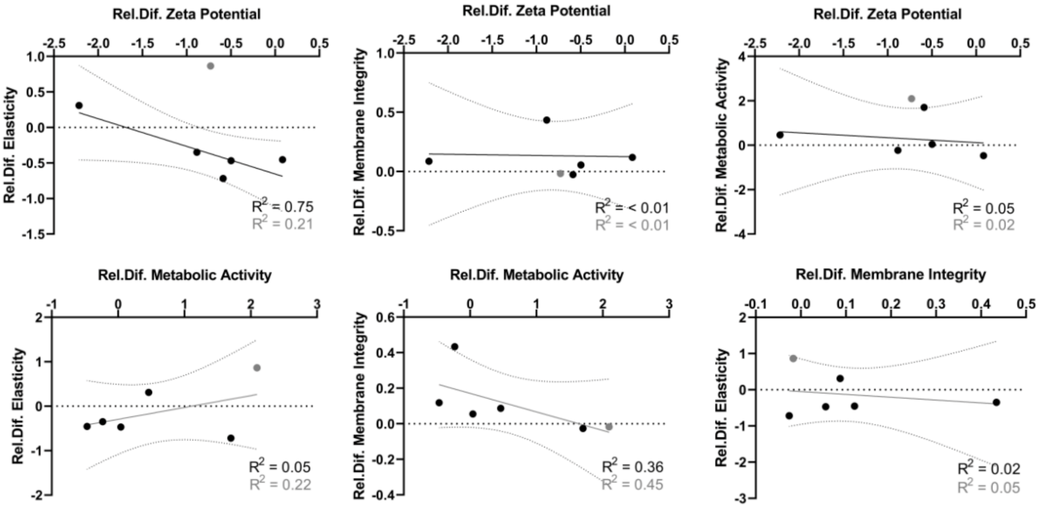

Figure S2. Linear regression analysis between different cellular parameters across species. Each black dot represents the relative difference of respective values in PBS to RTF buffer for one species. Values for *S. oralis* are shown as grey dots. Linear regression fit is shown with 95 % confidence interval and the corresponding correlation coefficient R2. For black lines and R2, *S. oralis* was excluded from calculation. Grey lines and R2 also include the grey values of *S. oralis*.

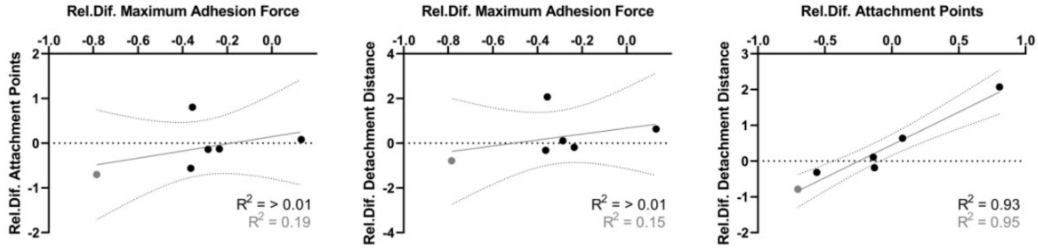

Figure S3. Linear regression analysis between different adhesion force parameters across species. Each black dot represents the relative difference of respective values in PBS to RTF buffer for one species. Values for *S. oralis* are shown as grey dots. Linear regression fit is shown with 95 % confidence interval and the corresponding correlation coefficient R^2. For black lines and R^2, *S. oralis* was excluded from calculation. Grey lines and R^2 also include the grey values of *S. oralis*.
